# Perinatal asphyxia and hypothermic treatment from the endocrine perspective

**DOI:** 10.3389/fendo.2023.1249700

**Published:** 2023-10-20

**Authors:** Nicola Improda, Donatella Capalbo, Antonella Poloniato, Gisella Garbetta, Francesco Dituri, Laura Penta, Tommaso Aversa, Linda Sessa, Francesco Vierucci, Mariarosaria Cozzolino, Maria Cristina Vigone, Giulia Maria Tronconi, Marta del Pistoia, Laura Lucaccioni, Gerdi Tuli, Jessica Munarin, Daniele Tessaris, Luisa de Sanctis, Mariacarolina Salerno

**Affiliations:** ^1^ Department of Translational Medical Sciences, Paediatric Endocrinology Unit, University “Federico II”, Naples, Italy; ^2^ Department of Emergency, Santobono-Pausilipon Children’s Hospital, Naples, Italy; ^3^ Department of Mother and Child, Paediatric Endocrinology Unit, University Hospital “Federico II”, Naples, Italy; ^4^ Neonatal Intensive Care Unit, San Raffaele University Hospital, Milan, Italy; ^5^ Pediatric and Neonatal Unit, San Paolo Hospital, Civitavecchia, Italy; ^6^ Department of Pediatrics, University of Perugia, Perugia, Italy; ^7^ Department of Human Pathology of Adulthood and Childhood, University of Messina, Messina, Italy; ^8^ Maternal and Child Department, Neonatal Intensive Care Unit (NICU) of University Hospital San Giovanni di Dio e Ruggi d’Aragona, Salerno, Italy; ^9^ Pediatric Unit, San Luca Hospital, Lucca, Italy; ^10^ Department of Pediatrics, Santa Maria Delle Croci Hospital, Ravenna, Italy; ^11^ Endocrine Unit, Department of Pediatrics, University Hospital San Raffaele, Milan, Italy; ^12^ Division of Neonatology and Neonatal Intensive Care Unit (NICU), Department of Clinical and Experimental Medicine, Santa Chiara University Hospital, Pisa, Italy; ^13^ Pediatric Unit, Department of Medical and Surgical Sciences of the Mother, Children and Adults, University of Modena and Reggio Emilia, Modena, Italy; ^14^ Pediatric Endocrinology Unit, Regina Margherita Children’s Hospital, Turin, Italy; ^15^ Department of Public Health and Pediatric Sciences, University of Turin, Turin, Italy

**Keywords:** perinatal asphyxia, hypothermic treatment, stress adaptations, endocrine sequelae, electrolytes disturbances, glucose abnormalities

## Abstract

**Introduction:**

Perinatal asphyxia is one of the three most important causes of neonatal mortality and morbidity. Therapeutic hypothermia represents the standard treatment for infants with moderate-severe perinatal asphyxia, resulting in reduction in the mortality and major neurodevelopmental disability. So far, data in the literature focusing on the endocrine aspects of both asphyxia and hypothermia treatment at birth are scanty, and many aspects are still debated. Aim of this narrative review is to summarize the current knowledge regarding the short- and long-term effects of perinatal asphyxia and of hypothermia treatment on the endocrine system, thus providing suggestions for improving the management of asphyxiated children.

**Results:**

Involvement of the endocrine system (especially glucose and electrolyte disturbances, adrenal hemorrhage, non-thyroidal illness syndrome) can occur in a variable percentage of subjects with perinatal asphyxia, potentially affecting mortality as well as neurological outcome. Hypothermia may also affect endocrine homeostasis, leading to a decreased incidence of hypocalcemia and an increased risk of dilutional hyponatremia and hypercalcemia.

**Conclusions:**

Metabolic abnormalities in the context of perinatal asphyxia are important modifiable factors that may be associated with a worse outcome. Therefore, clinicians should be aware of the possible occurrence of endocrine complication, in order to establish appropriate screening protocols and allow timely treatment.

## Introduction

Perinatal asphyxia (PA) is defined as critical reduction in the oxygenated blood supply to the fetus that occurs around the time of birth because of a variety of events, including maternal or fetal hemorrhage, intermittent or acute umbilical cord compression, uterine rupture, or dystocic delivery. PA represents one of the three most important causes of neonatal morbidity and mortality (1).

Most asphyxiated babies recover successfully from the hypoxic insult, but some patients experience permanent damage of both vital and non-vital organs, especially the brain, heart, kidney, and lungs. Hypoxic brain damage may result in hypoxic ischemic encephalopathy (HIE), which has an incidence of 1−8 per 1000 live births in developed countries and is the most common cause of long-term disability in full-term infants (1, 2). The endocrine system plays a critical role in coordinating metabolic, respiratory and vasomotor responses to hypoxia (3). Moreover, in a small but not negligible percentage of cases, PA may be associated with endocrine dysfunctions including electrolytes and glucose disturbances, adrenal insufficiency (AI), thyroid hormone abnormalities, and damage to the pineal gland.

Hypothermia treatment (HT) represents the standard treatment for near-term infants with moderate-to-severe HIE (4). Despite leading to a clinically relevant reduction in major neurodevelopmental disability and cerebral palsy (CP), hypothermia is only partially effective and may in turn cause organ damage leading to endocrine disturbances (4–6). HT has become routine care relatively recently and while its effects on the nervous, cardiopulmonary, and renal systems have been thoroughly investigated (7–9), the endocrine and metabolic effects have not yet been sufficiently considered.

Endocrine alterations during PA represent important modifiable factors that can be associated with increased mortality, and worse neurodevelopmental outcome, and therefore clinicians need to be aware of their possible occurrence. Screening strategies for endocrine complications are essential to ensure timely diagnosis and therapeutic intervention as well as to improve neuroprotection.

Aim of this review is to summarize the main studies evaluating the effects of perinatal asphyxia and of HT on the endocrine system, with a focus on the pathogenic mechanisms, monitoring and treatment strategies of asphyxiated children.

## Methods

The authors focused their search on pathogenic mechanisms, diagnosis and treatment of the most relevant endocrine consequences of PA and HT. Literature search was performed in PubMed by using selected keywords: ‘perinatal asphyxia’ OR ‘hypoxic ischemic encephalopathy’ OR ‘hypothermia’ AND ‘neonates’ AND ‘adrenal’ OR ‘thyroid’ OR ‘sodium abnormalities’ OR ‘electrolyte abnormalities’ OR ‘diabetes insipidus’ OR ‘hypocalcemia’ OR ‘hypercalcemia’ OR ‘gonads’ OR ‘hypoglycemia’ OR ‘hyperglycemia’ OR ‘pituitary function’ OR ‘pineal gland’. Moreover, a manual search for additional relevant publications was made of the bibliographies of the papers identified automatically.

All authors independently identified the most relevant papers published in English, including original papers, metanalysis, clinical trials, reviews, and case series. Given the narrative nature of the review, the formulation of recommendations regarding the management of each specific condition was based on the authoritativeness and expertise of its authors, in the context of existing literature. The individual contributions were collected and critically reviewed by all authors, who also gave their approval on the final version.

## Pathophysiology of perinatal asphyxia and the rationale for hypothermic treatment

PA is characterized by interruption of gas exchange with subsequent hypoxia, hypercarbia, and acidosis (1) occurring before, during, or after labor in the perinatal period. It may be related to maternal, placental, umbilical cord and neonatal causes, including uterine rupture, preeclampsia, placental abruption, cord compression, intrauterine pneumonia, severe meconium aspiration, cardiac or pulmonary diseases, dystocic delivery and medication effects (2).

When the oxygen supply is compromised, fetal blood flow is initially redistributed to vital organs (brain, heart, and adrenal glands), with detriment of other organs (2). Centralization of blood flow is driven by carotid chemoreceptors activation during hypoxic-ischemic injury causing massive catecholamine release with peripheral vasoconstriction. Despite this adaptive process, prolonged hypoxia causes cerebral anaerobic metabolism causing lactate production and metabolic acidosis (1). Similarly, in case of prolonged hypoxemia, cardiac output fails to maintain myocardial oxygenation resulting in metabolic acidosis, myocardial failure, and shock (9). Moreover, nephron activity is depressed and kidneys show elevated susceptibility to reperfusion injury, resulting in decreased excretory function, with both electrolytes and pH imbalance (1, 2). Other non-endocrine organs possibly affected by hypoxic injury are the liver, with hyper-transaminasemia and coagulopathy, and the lungs with pulmonary hypertension and hemorrhage (1, 2).

Hypoxic brain damage mainly results in HIE, which is the most common cause of long-term disability in full-term infants.

The pathogenesis of neonatal HIE involves an early phase of energy failure, followed after at least six hours by reoxygenation and reperfusion injury, with depletion of the antioxidant defense system due to oxidative stress and subsequent tissue damage (10). The severity of HIE is commonly classified as mild, moderate or severe according to the Sarnat grading, which correlates with the degree of neuronal damage and is predictive of adverse neurodevelopmental outcomes (6).

The interval between the two pathophysiologic phases represents the therapeutic window for HT in infants with moderate-to-severe HIE. Such treatment has been shown to reduce mortality or the risk of long-term neurodevelopmental disability (4), by reducing cerebral metabolism and by attenuating pro-inflammatory pathways that lead to necrosis and neuronal apoptosis, including the release of excitatory amino acids, and the production of free radicals and nitric oxide (11). However, in severe cases, despite maximal care, only little improvement is observed, with important repercussions on the family, health care system, and society. Indeed, when the severity or duration of the neuronal insult exceeds the capacity of the CNS to repair the damage, depending upon susceptibility characteristics that the genome confers on neuronal tissue, inflammation persists and the damaged brain tissues lose the support of neurotrophic factors (12, 13). In this respect, experimental evidence in animal and/or human models indicate that more prolonged administration of additional treatments, including growth factors, stem cells, antioxidants, substances reducing excitotoxicity, local inflammation, and anti-apoptotic agents may improve the therapeutic efficacy of HT, by preventing more severe neuronal and synaptic injury and potentiating repair and regeneration of the damaged brain tissue (12–14).

Eligibility criteria for HT are: a) gestational age of at least 35 weeks and weight at least 1.8 kg b) less than 6 hours of life, c) asphyxia as defined by the presence of at least two of the following: Apgar score less than 6 at 10 minutes or persistent need for resuscitation at 10 minutes, any acute perinatal event associated with cordon arterial pH <7.0 or base excess≤ -12 mmol/L obtained within the first hour of life, and d) moderate/severe HIE according to Sarnat staging (15). HT is not recommended in case of oxygen requirement greater than 80%, major congenital abnormalities, severe uncontrolled coagulopathy or low probability of survival (15).

The HT protocol consists of 72 hours of hypothermia (core temperature around 33.5°C), followed by a gradual rewarming phase (15). All children undergoing HT should perform a brain MRI within the first month of life which allows for early recognition of cerebral abnormalities and helps predict neurodevelopmental outcomes (16).

## Endocrine effects of perinatal asphyxia

**Figure 1:**
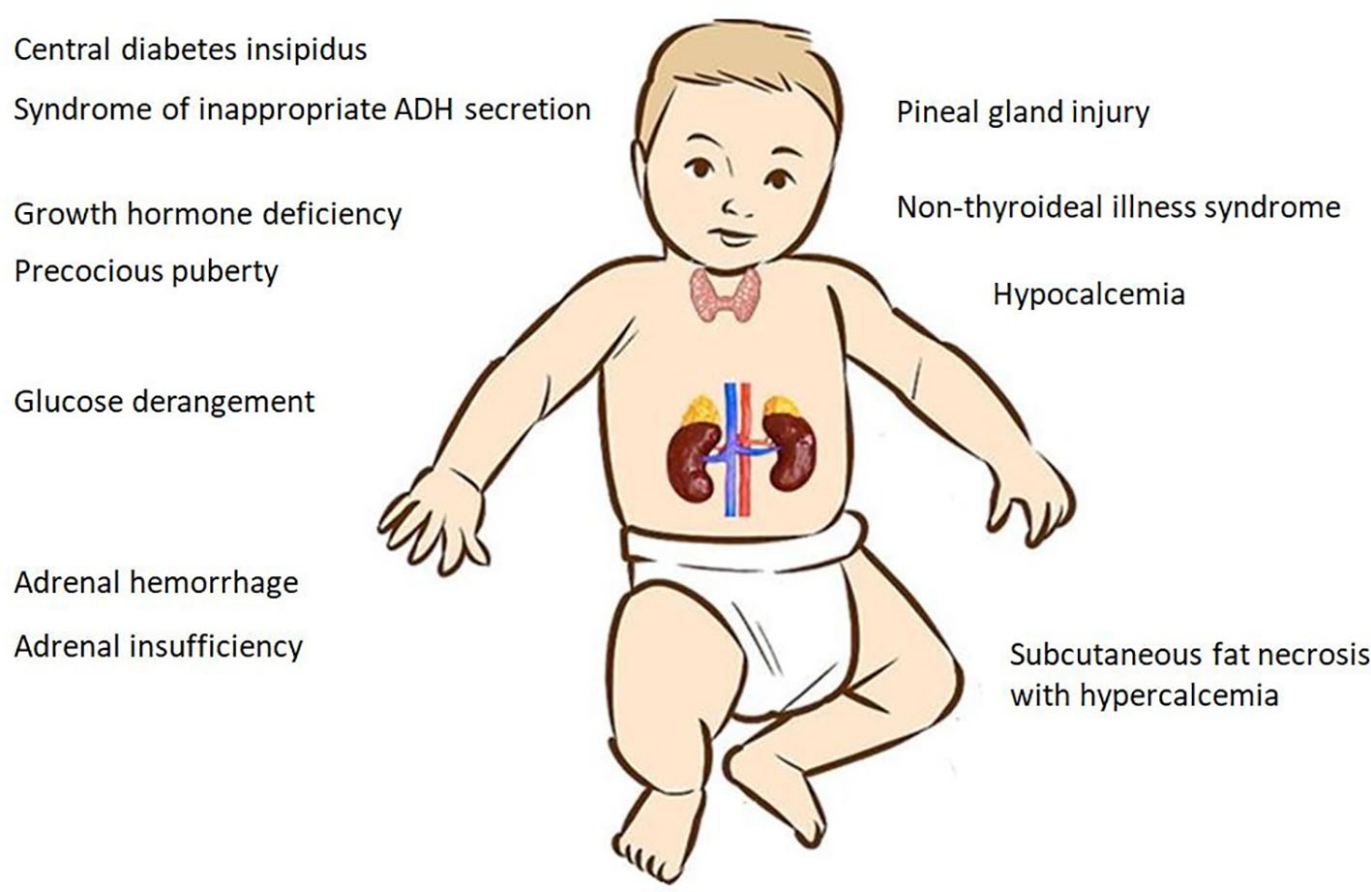
Endocrine manifestations of birth asphyxia.

The clinical spectrum of PA encompasses several endocrine manifestations that can lead to acute decompensation and even life-threatening events (**Figure 1**). The variability of the clinical presentation depends on several factors, such as gestational age, the severity and/or duration of hypoxia, and the use of HT (17, 18).

### Glucose homeostasis

Oxidative metabolism accounts for almost all glucose uptake by the brain (19). Under hypoxic conditions, excess lactate serves as a substrate for gluconeogenesis, which is in turn stimulated by the release of glucocorticoids and catecholamines (20, 21). The brain increases utilization of glucose and reactive vasodilation increases the glucose availability for anaerobic glycolysis; nevertheless, the worsening acidosis is ultimately associated with impaired cardiac function, decreased glucose release, loss of cerebrovascular autoregulation, and depletion of local glucose storage. The neonatal brain compensates initially by lowering cerebral energy requirements and enhancing the ability to utilize lactate as an alternative energy source; however, asphyxia leads to a failure of compensatory mechanisms with impaired antioxidant system and worsening of encephalopathy (19, 20).

Both hypoglycemia and hyperglycemia are known to occur more frequently in high-risk categories of newborns, including asphyxiated neonates (22, 23). In fact, almost half of infants receiving HT for HIE may experience at least one episode of hypoglycemia, with the first episode usually occurring within the first 24 hours, and a progressive reduction in the frequency over the following days (23–25). Symptoms of neuroglycopenia in the neonatal period are highly non-specific and may include lethargy, cyanosis, irregular breathing, hypotonia, irritability, abnormal cry, feeding problems, seizures, myoclonic jerks, coma, and apnea. Thus, they can easily be masked by the symptoms of HIE (22).

The pathogenesis of hypoglycemia in PA is multifactorial involving severe glycogen depletion secondary to catecholamine release, reduced glucagon response, increased insulin release, and reduced adiponectin (which promotes insulin sensitivity) (23, 26–28). In response to increased insulin signaling, activation of the insulin receptor (IR) results in increased expression of lipogenic genes, and inhibition of the expression of gluconeogenic genes, through coordinated activation of specific transcription factors, such as cyclic AMP-responsive element-binding protein (CREB), Forkhead box O (FOXO), and Sterol regulatory element-binding protein 1 (SREBP1) (29). While stimulating effects of insulin on lipogenesis are largely mediated by SREBP1 and CREB, IR-mediated phosphorylation of FOXO leads to the exclusion of the protein from the nucleus, with reduced transcription of genes involved in gluconeogenesis and glycogenolysis (30),. Signaling through CREB and FOXO is also crucial for β-cell survival as well as for insulin gene transcription, and glucose-mediated insulin exocytosis (29, 30). These processes are disrupted in the context of perinatal asphyxia, due to the marked sensitivity of pancreatic β cells to oxidative stress agents. In fact, under hypoxic conditions, activation of hypoxia-inducible factors stimulates anaerobic glycolytic flux independently from blood glucose concentrations, resulting in higher insulin and lower glucose concentrations in fasting conditions, and impaired insulin response to postprandial hyperglycemia (31). In a few cases, inappropriate insulin secretion becomes clinically relevant to require specific medical treatment (26) (**Table 1**). Indeed, a recent retrospective study showed that PA and greater than standard resuscitation accounted for 3% and 33% of the causes of perinatal stress-induced hyperinsulinemic hypoglycemia, respectively (26).

**Table 1:** Acute management and follow-up of major endocrine features.

	Pathogenic mechanisms	Management	Follow-up
**Hypoglycemia (** 32 **–** 33)	Increased or inappropriate insulin secretion, delayed feeding, depletion of glycogen stores, reduced counter-regulatory response	iv bolus 2 ml/kg 10% dextrose, followed by glucose delivery 5-6 mg/kg/min In the absence of iv access, rescue SC or IM glucagon bolus 100-200 mg/kg/dose *Hyperinsulinism* Start diazoxide 5-20 mg/kg/day orally, with incremental doses eventually combined with chlorothiazide.	Monitor plasma glucose levels regularly, especially during HT and weaning from TPN Modify glucose supply according to glucose monitoring (if the osmolality of the infused solution is >600 mOsm/L, place a central line) Monitor for side effects of diazoxide and try to discontinue when appropriate
**Hyperglycemia (** 34, 35)	Interventions aimed at increasing glucose levels, hepatic and pancreatic islet dysfunction	Maintain optimal hydration to counteract osmotic diuresis Decrease glucose supply up to a minimum of 3-4 mg/kg/min If blood glucose persistently >180 mg/dl despite reduction in glucose supply, or the neonate has signs of plasma hyperosmolarity, consider starting sc or iv insulin as a continuous infusion or in boluses 0.05-0.1 UI/kg/dose every 4-6 hours Insulin infusion rate adjustments of 0.01 U/kg/h Withdraw insulin if plasma glucose levels persistently <15 mg/dl	Monitor blood glucose initially every 30 minutes and then hourly Check for rebound hyperglycemia, after insulin discontinuation Monitor for sodium and potassium abnormalities, especially during insulin infusion
**Hyponatremia (** 36–39)	Acute brain injury, acute kidney damage, SIADH, fluid overload, hypothyroidism, hypocortisolism	Fluid restriction 50-70 ml/kg/day in the first 24h of life with further increase by 10 ml/kg/day Evaluate and replace concomitant overt hypothyroidism or hypocortisolism Consider vaptans if persistent hyponatremia or challenging management of fluid restriction	Water balance and hydration status monitoring Electrolytes monitoring every 8 hours over the first 24-48 hours
**Hypernatremia (** 38, 39)	Severe brain damage leading to central diabetes insipidus	Avoid Hypotonic solutions Initial dose of Desmopressin at 1 mcg/kg/day, then adjust the dose according to water balance and electrolytes	Water balance monitoring Electrolytes monitoring every 8 hours over the first 24-48 hours and then regularly
**Hypocalcemia (** 40, 41)	Altered PTH response or increased calcitonin, increased phosphate or bicarbonate load, low calcium intake, acute renal injury	*Mild, asymptomatic hypocalcemia* If possible, oral supplementation of 10% calcium gluconate calcium gluconate 10% iv at 1.5-2 ml/kg continuously or in divided doses every 6-8 hours *Severe, symptomatic hypocalcemia* calcium gluconate 10% iv infusion at 0.5-1 ml/kg slowly in 10 min, followed by calcium gluconate 10% iv at 1.5-2 ml/kg every 6-8 hours	Calcium measurements every 6-8 hours, especially during and after weaning calcium supplements Cardiac monitoring Try to gradually discontinue calcium supplementation if serum calcium persistently stable
**Hypercalcemia (** 42–44)	SFN secondary to HT and/or traumatic delivery	*Mild, asymptomatic hypercalcemia* - Discontinue Vitamin D - Low-calcium formula - If persistent hypercalcemia, consider glucocorticoids *Severe hypercalcemia (>12 mg/dl) or overt symptoms* - Hospital admission - Discontinue Vitamin D - Low-calcium formula or parenteral nutrition - iv hydration with saline and then furosemide infusion - Consider subcutaneous Calcitonin - If persistent hypercalcemia, consider iv Pamidronate, one or more doses	Maintain adequate hydration Regular monitoring of serum and urine calcium and urine calcium/creatinine ratio, especially after dietary changes ECG monitoring Repeated abdomen US, looking for signs of nephrocalcinosis
**Primary adrenal** **insufficiency (** 45–47)	Adrenal hemorrhage, dystocic delivery, macrosomia, fetal acidemia	Hydrocortisone 50-100 mg/m2, followed by 50-100 mg/m2/day, iv, as a continuous infusion or divided in 4 doses. When oral intake is possible, 10-12 mg/m^2^/day oral hydrocortisone, in 3 daily doses Consider oral fludrocortisone if persistently low sodium, high potassium, and high serum renin concentrations	Strict clinical monitoring (fluid balance, blood pressure, blood glucose) Frequent electrolytes monitoring Try to discontinue hydrocortisone and fludrocortisone if adrenal lesions resolve in US follow-up and clinical and biochemical parameters are stable.
**Hypotension refractory** **to fluids and amines (** 48, 49)	Relative adrenocortical insufficiency	Hydrocortisone iv 1 mg/kg bolus test. In case of improvement of cardiovascular parameters, continue 1 mg/kg hydrocortisone every 8-12 hours for 3-5 days	Monitor blood glucose and urinary output

ECG, electrocardiogram; HT, hypothermic treatment; iv, intravenous; PTH, parathyroid hormone; SC, subcutaneous; SFN, subcutaneous fat necrosis; SIADH, syndrome of inappropriate antidiuretic hormone; TPN, total parenteral nutrition; US, ultrasound.

Hyperglycemia has also been reported in up to 50% of the neonates with encephalopathy (7). Parmentier et al. showed that 35.4% of 223 infants receiving HT had hypoglycemia, which was severe in 22.4% (19). In this study 80% of the infants with hypoglycemia also had later episodes of hyperglycemia, which might result either from interventions aimed at increasing glucose levels or from hypoxia-related hepatic and pancreatic islet dysfunction (19, 25).

Several studies have shown that hypoglycemic episodes correlate with the severity of both asphyxia and HIE (20, 50) and that glucose instability is predictive of adverse neurodevelopmental outcome in neonates with HIE (25, 51–53). Indeed, Montaldo et al. (25) reported that 35% of infants with unfavorable outcome had out-of-range glucose values, compared with only 18% in the group with favorable outcome; moreover, longer duration of hypoglycemia and greater area under the hypoglycemic curve were associated with adverse neurodevelopmental outcomes at 18-24 months. Similarly, Basu et al. reported that infants with HIE who experienced hypoglycemia or any glucose derangement during the early postnatal period had a 3- to 6-fold increased risk of unfavorable outcomes (death or severe neurodevelopmental disability at 18 months) compared with normoglycemic infants (50). In a recent study, hypoglycemia predicted lower motor and cognitive scores in preschool age (19), after adjustment for severity of HIE.

Conversely, Pinchefsky et al. documented that in neonates with HIE on 3-days Continuous Glucose Monitoring (CGM), periods of hyperglycemia, but not of hypoglycemia, were associated with worse background EEG scores, reduced sleep–wake cycling, and seizures (54). Similarly, in a more recent study, Kamino et al. found that maximum glucose concentrations over the first 48 hours, but not minimum, predicted basal ganglia and watershed injury in neonates suffering from HIE (55). Glucose concentrations above 10.1 mmol/L during the first 48 hours of life predicted higher composite outcome of severe disability or death, higher Child Behavior Checklist T-scores, worse neuromotor score, and higher risk of cerebral palsy at 18 months of life (55).

A growing body of evidence suggests that glucose derangement (especially hypoglycemia) makes certain areas of the brain more vulnerable to hypoxic–ischaemic injury. While neonatal hypoglycemia has classically been linked to parieto-occipital injury, the pattern of hypoglycemia-related injury in neonates with HIE seems to include involvement of the corticospinal tract, the basal ganglia, the sensorimotor cortex, and watershed areas (23). In a more recent study of neonates with HIE undergoing HT, even higher peak glucose concentrations on day one of life were associated with changes in MRI spectroscopy in many areas other from those associated with hypoglycemia (anterior and posterior white matter, corpus callosum, lentiform nucleus, pulvinar, and optic radiations) (56). So far, no clear-cut explanation has been found for the susceptibility to dysglycemia of these specific areas of the brain in asphyxiated neonates. Various mechanisms have been proposed, including altered patterns of regional perfusion, hypoglycemia-induced excitatory neurotoxins active at cell-type-specific N-methyl-D-aspartate receptors, increased mitochondrial free radical generation and initiation of apoptosis (57). Additional hypotheses may be immaturity of the white matter in some infants, and reduced myelin fiber formation, due to inhibited proliferation, migration, and differentiation of oligodendrocyte precursors and accelerated oligodendrocytes apoptosis induced by hypoglycemia (58).

These observations indicate that proactive avoidance of glucose instability is a neuroprotective strategy in the context of neonatal encephalopathy (25, 32). The Pediatric Endocrine Society advises that a “safe target” during the first 48 hours should be close to the mean for healthy newborns and above the threshold for neuroglycopenic symptoms (50 mg/dl; 2.8 mmol/L) (59). This can be obtained through regular monitoring of glucose levels every 4 to 6 hourly during HT and within the 48 hours of warming, together with adequate energy supply, via enteral or parenteral route (32, 59) and correction of hypoglycemia (32, 33, 59) and hyperglycemia episodes (34, 35) (**Table 1**). However, reference ranges in healthy full-term newborns may not be appropriate in infants at risk of impaired metabolic adaptation, as individual susceptibility to brain injury can vary depending on comorbid conditions and an infant’s ability to produce and use alternative substrates (32).

Despite being considered the gold standard in infants, intermittent glucose monitoring might not detect low glucose concentrations or conversely might detect only temporary hypoglycemia, leading to unnecessary treatment. CGM has the advantage of tracking glucose levels continuously, potentially improving clinical management. However, the accuracy and functioning of sensors during cooling remains to be determined (25). In all patients who have recurrent episodes of hypoglycemia during weaning from parenteral nutrition, or higher than normal glucose requirements to maintain euglycemia (>8 mg/kg/min), evaluation of insulin concentrations at the time of hypoglycemia is mandatory (33). Incremental introduction of enteral feeds by orogastric tube during HT and rewarming period, possibly with maternal breast milk, is safe and may be beneficial for glucose metabolism, for gut microbiome early stabilization and may also have a neuroprotective effect (59). Non-nutritive enteral feeding (10 ml/kg/day) can be started immediately during HT with small increases, if well tolerated. At the end of hypothermia, oral feeding can be re-established with caution, if suction is good, otherwise the orogastric tube should be continued (59).

### Fluid and electrolytes balance

#### Syndrome of inappropriate antidiuretic hormone release

A marked increase in serum ADH and copeptin has been reported after vaginal delivery, triggered by activation of the hypothalamic-pituitary axis and the sympathetic nervous system in response to stress (60). Copeptin is the C-terminal part of preproADH, released in a 1:1 ratio. Its *in vitro* stability makes it an ideal surrogate marker of peripheral ADH release (60). The reasons for this increase are unknown, but ADH seems to exacerbate brain edema, vasoconstriction, disruption of the brain blood barrier, and neuroinflammation during ischemic brain injuries (61).

Newborns with HIE are at risk of developing hyponatremia due to acute kidney injury, overload of administered fluids, SIADH, urinary sodium loss related to decreased tubular sodium reabsorption or treatments such as hypothermia (62, 63). Hypothyroidism and hypocortisolism may contribute to hyponatremia especially in premature newborns (64, 65). Multifactorial SIADH may occur in newborns with HIE due to hypoxic brain injury, pain, vomiting or drugs with ADH-like effect (especially anticonvulsivants). Strict monitoring of fluid balance and daily electrolyte assessment (every 8 hours in the first 24-48 hours) evaluation is mandatory in neonates affected by HIE (36).

In the case of hyponatremia, firstly, the evaluation of fluid administration rate and sodium content is necessary. The biochemical evaluation should include measurement of TSH, fT4, ACTH, cortisol, serum osmolality, urine osmolality, urinary sodium and, if available, serum copeptin.

SIADH should be considered in hyponatremia associated with low serum osmolality (<275 mOsm/kg), urine osmolality higher than 100 mOsm/kg, urinary sodium higher than 40 mmol/L, low urinary output and, when available, copeptin inappropriate for serum osmolality (generally above than normal range) (37).

Fluid restriction should be considered as first choice in case of SIADH and 50-70 ml/kg/day is initially considered appropriate for neonates in the first day of life and a further increase should be evaluated daily by 20 ml/kg/day for each subsequent day (37, 38, 66) (**Table 1**). Fluid restriction is often difficult to obtain or maintain and may always not be effective.

Under these conditions or when SIADH becomes chronic with potentially severe neurological symptoms, a class of aquaretic agents called vaptans can be used (39) to drive electrolyte-free polyuria. Low-dose titration approach should be used at treatment initiation and subsequent dose modulation should be performed based on monitoring of serum sodium and urinary output. Fluid restriction should gradually turn towards normal daily fluids intake. Vaptans are still considered off-label in pediatric age in both Europe and USA, and in addition to monitoring fluid and electrolyte balance, liver enzymes assessment during treatment is also required. Given the negative effects played by ADH during ischemic brain injury, a role has been proposed for Vaptans to mitigate neurological consequences of ischemic stroke in adults (67). Rapid corrections of hyponatremia should be avoided. The correction rate should be of 4-6 mmol/L in the first 4-6 hours, 10-12 mmol/L in the first 24 hours, <18 mmol/L in the first 48 hours (37). Finally, in the presence of concomitant biochemical features of overt hypothyroidism or AI, the decision to initiate treatment should be considered on a case-by-case basis (37).

#### Central diabetes insipidus

HIE has been associated with central diabetes insipidus (CDI) in anecdotal cases (67, 68). The supraoptic and paraventricular hypothalamic nuclei are resistant to hypoxia and reduced blood supply due to the presence of many neurosecretory cells, more than 90% of which should be destroyed before CDI occurs. Moreover, the posterior pituitary gland receives its blood supply by the inferior hypophyseal artery which functions under high pressure and is preserved by low-pressure or hypoxic damage (69). Finally, glucocorticoid-dependent mechanisms of brain tolerance to hypoxia and HT-related decreased energy demand may further preserve hypothalamic nuclei from hypoxic damage (70). Thus, only severe hypoxic/ischemic insults can interfere with the integrity of ADH release (67, 68). In case of hypernatremia, firstly, fluid balance and sodium content of the administered fluid should be carefully evaluated. Biochemical assessment requires measurement of serum and urine osmolality and, if available, a serum sample for copeptin. Hypernatremia, high serum osmolality (associated with low copeptin level), low urinary osmolality and high urinary output are diagnostic for CDI (39). During hypernatremia correction, hypotonic solutions should be avoided and the daily sodium intake should be provided. Desmopressin administration should be started at the lowest dose possible (1 mcg/kg/day), with further dose adjustments based on fluid balance and serum sodium levels (**Table 1**) (39).

#### Hypocalcemia

Hypocalcemia, defined as total serum calcium (Ca) level <4 mEq/L in term newborns and <3.5 mEq/l in preterm newborns or ionized serum Ca level <2.0 mEq/l in newborns at term and <1.75 mEq/l in preterm newborns, is common in asphyxiated children, and is often associated with other electrolyte disturbances, such as hyponatremia, hyperkalemia, hypomagnesemia, and even hyperphosphatemia (71). Hypocalcemia was also reported in 17% of the newborns undergoing HT, even though, in contrast to what was observed for hyponatremia, it seems that implementation of HT has led to a reduction in incidence despite lower Ca intakes, suggesting positive effects of hypothermia on Ca metabolism (40).

Asphyxia-related hypocalcemia is explained by several possible mechanisms, including a slow PTH secretory response by the parathyroids to the postnatal fall in plasma Ca concentration, an increased phosphate load due to tissue catabolism or excess parenteral supply, low Ca intake due to delayed feeding, excess bicarbonate therapy, renal failure and finally increased calcitonin concentrations (62, 71). While for hyponatremia and hypokalemia several studies have indicated a close correlation with the severity of asphyxia, for hypocalcemia the results are still conflicting. Some authors have failed to find a correlation between hypocalcemia and severity of hypoxic-ischemic encephalopathy, while others have reported that severely asphyxiated children are more prone to develop severe Ca impairments requiring prompt medical intervention (41).

Symptoms of hypocalcemia are non-specific and can be masked by the asphyxiated condition, being related to alterations of neuromuscular and CNS activity (irritability, agitation, apnoea, lethargy with poor sucking, seizures), and of cardiac rhythm (arrhythmia with even increased risk of sudden death).

Ca flow inside neurons and glial cells due to glutamate-mediated excitotoxicity results in activation of calcium-dependent lytic enzymes, oxidative stress, mitochondrial dysfunction, cytotoxic edema, and apoptosis (6). Thus, serial evaluations of ionized Ca, the biologically active fraction of Ca, are required to adequately diagnose hypocalcemia, avoiding unnecessary or prophylactic calcium administration (62).

Acute treatment of symptomatic hypocalcemia involves the use of intravenous (iv) 10% Ca gluconate at a dose of 100 mg/kg (1 mL/kg) infused slowly, over 10-20 minutes, under close ECG monitoring to avoid arrhythmias (72, 73) (**Table 1**). Alternatively, iv Ca chloride (20 mg/kg or 0.2 mL/kg) can be given, a more rapidly metabolized preparation that may be preferable in life-threatening situations. In case of symptoms persisting after the initial dose, the dose of Ca can be repeated after 10 minutes. After acute treatment, Ca gluconate maintenance can be administered at an iv dose of 100 mg/kg (1 mL/kg) elemental Ca daily (72, 73). If enteral feedings are tolerated, oral Ca glubionate can be given at a dose of 30-50 mg/kg/day in four divided doses, although its high osmolality and sugar content may cause gastrointestinal irritability or diarrhea. Alternatively, 10% Ca gluconate (up to 500 mg/kg/day) can be used and divided over four-six feedings (72, 73). Serum Ca concentrations usually improve within 1-3 days of treatment; Ca supplements should be withdrawn gradually, when serum Ca levels have normalized, and the newborn is able to feed sufficiently for needs (74).

To enhance Ca absorption, vitamin D3 at 400-800 IU/day should be added, depending on the gestational age and vitamin status of the neonate and the mother. Calcitriol at a dose of 0.08 to 0.1 mcg/kg/day may represent an alternative therapy, for possible hepatic or renal failure or immaturity. In case of concomitant hypomagnesemia, the latter should be treated before correcting the hypocalcemia, with 50% Magnesium sulfate at a dose of 50-100 mg/kg (0.4-0.8 mEq/kg/day) divided in 2 doses, iv over at least 2 hours or intramuscular (IM), until serum magnesium concentration is >1.5 mg/dL (0.62 mmol/L) (72, 73). In infants with associated hyperphosphatemia, breast milk is preferable for its correct Ca/P ratio; alternatively, a low phosphate formula should be used, even though the differences in phosphate concentrations among various formulas are small and may not be clinically significant (72, 73).

#### Hypercalcemia

Hypercalcemia occurs rarely in asphyxiated newborns and is most often iatrogenic. Indeed, hypercalcemia can be encountered in HT, mainly associated with subcutaneous fat necrosis (SFN) (75), even though this condition may be normocalcemic or even hypocalcemic in a much lower percentage of cases, due to an immature PTH response (76). The median onset of SFN is around day 6 of life, but it has been reported up to 270 days (76). Its incidence has decreased over time because of improved skin care (42) and is currently estimated around 1% of cases undergoing HT (4, 75). Neonatal fat has a relatively high concentration of saturated fatty acids (palmitic and stearic acids), with a high melting point predisposing the adipose tissue to crystallization during hypothermia. Other possible mechanisms contributing to SFN are hypoxic damage, mechanical pressure with subsequent worsening of hypoperfusion, and localization of brown adipose tissue in specific sites (76). Clinically, it is characterized by multiple indurated plaques or nodules, with or without erythema on the cheeks, posterior trunk, buttocks, and limbs (43, 76).

Hypercalcemia occurs, usually within the first month, in 36-56% of affected neonates and it may be life-threatening. It is likely due to extrarenal production of 1,25- dihydroxyvitamin D3 by inflammatory skin cells expressing high levels of 1-alpha hydroxylase. Alternatively, direct release of calcium from the skin lesions has been suggested (76).

Only 50% of these neonates show classic symptoms of hypercalcemia (poor feeding, vomiting, failure to thrive, constipation, muscular hypotonia, lethargy, irritability, convulsions, hypertension); routine screening for hypercalcemia is therefore recommended for neonates with or at-risk of developing SFN (76).

SFN is a self-limiting panniculitis; however, when complicated by hypercalcemia, several treatment options are indicated on a case-by-case basis, along with ECG monitoring for possible arrhythmias (**Table 1**) (43). First, iv hyper-hydration, together with low Ca formula, avoiding vitamin D supplementation. A Ca-losing diuretic, such as furosemide, and/or corticosteroids represent the next step, in case of persistent hypercalcemia. Finally, bisphosphonates have recently been proposed as first-line treatment in symptomatic newborns (especially iv pamidronate in one or more doses of 0.25-0.5 mg/Kg) (43, 44, 76).

#### Prevention strategies of fluid and electrolytes disturbances

During HT there is a reduction in trans-epidermal water loss due to skin vasoconstriction, in the urinary output and in the respiratory water losses due to mechanical ventilation (77). The chances of fluid retention increase, therefore some authors recommend systemic fluids and sodium restriction to avoid hyponatremia (63). The current recommendations suggest that from birth and within the first 24 hours of HT, infusion should start with 40-50 ml/kg/day, adjusting fluid intake according to the fluid balance. It is recommended to start with an isotonic glycoprotein solution containing no sodium or potassium, but with addition of Ca (at maintenance value of 6 ml/kg/day).

However, it has been suggested that the systematic approach of fluid restriction during HT should be avoided in the absence of overt SIADH, to avoid further end-organ damage to kidneys and brain (38). On the other hand, fluid overload may worsen hyponatremia and lead to cerebral or pulmonary edema. Moreover, there is no evidence-based data that support or refute that the systematic fluid restriction approach following PA affects mortality or morbidity (77–79).

Electrolytes should be added after 24 to 48 hours, in the absence of severe dyselectrolytemia, when electrolytes and renal function are stable. It is recommended to avoid potassium supplementation during cooling, because of the risk of hyperkalemia during rewarming. Sodium, Potassium and Ca have to be checked every six hours during the 72 hours of HT, and every twelve hours during the 48 hours of warming (38).

Careful skin care by changing the neonate posture several times a day during HT has been suggested to reduce the risk of HT-related adiponecrosis (75). If a rigid mattress is used for systemic hypothermia, it may be helpful to place a sheet between the newborn’s mattress. Regular skin inspection is required in the first two months of life in children receiving HT or born from a traumatic delivery or shoulder dystocia, to identify late adiponecrosis or very small lesions (43, 76). Moreover, families should be informed of the possible occurrence of such lesions, as well as possible symptoms of hypercalcemia (especially vomiting and poor weight gain or feeding) (43). The concentrations of total and ionized Ca, together Ca/creatinine ratio in the urine, should be checked weekly in the first month after detection of the lesions, especially if they are large (76), and monthly thereafter or in case of symptoms of hypercalcemia within the following 6 months (43, 76). In those infants who develop hypercalcemia, determination of serum 1,25(OH)2D3 and parathyroid hormone (PTH) allows confirmation of PTH-independent hypercalcemia, while regular renal US monitoring is required to detect nephrocalcinosis (76).

### Adrenal gland

#### Adrenal hemorrhage and adrenal insufficiency

Neonatal adrenal hemorrhage (AH) occurs in up to 3% live births (45) and may be due to PA in a variable percentage of cases. In the largest series described so far (80), AH was more frequently associated with well-known risk factors for PA, such as vaginal delivery (95.9%), macrosomia (21.6%), and fetal acidosis (31%). In another study (81), among 37 cases of AH diagnosed over a 4-years period, 10.8% had HIE, while 10.8% and 18.9% were associated with traumatic delivery and the need for resuscitation soon after birth, respectively. The vulnerability of the neonatal adrenal gland to hemorrhage may be explained by its large size and peculiar vascular structure, characterized by a large arterial supply that drains into a few veins at the corticomedullary junction and eventually into a single adrenal vein with a thick muscle wall (46). AH during PA may result from a marked decrease in perfusion pressure causing ischemic necrosis of vessels at the corticomedullary junction or from reperfusion injury (82). Alternatively, AH may also be related to venous vasoconstriction and platelet aggregation favored by massive release of ACTH and/or catecholamines (82, 83), or to the marked increase in venous and arterial perfusion pressure associated with traumatic delivery. The latter factor may also explain the male prevalence of AH likely due to higher birth weight (45).

The right adrenal gland is involved in about 70% of cases, because the right adrenal vein flows directly into the inferior vena cava, making it more susceptible to venous pressure fluctuations or compression between the liver and spine (46). Bilateral AH accounts for about 10% of cases (80).

AH may be identified incidentally or be symptomatic (46). Symptoms are non-specific and may include paleness, feeding difficulties, vomiting, palpable abdominal mass, indirect jaundice, hypothermia, tachypnea, hypotonia, or lethargy. In three independent series (80, 81, 84) jaundice was the most common symptom, being reported in 50-85% of the cases. Conversely, Fedakar et al. found hypotonia and lethargy to be the most frequent symptoms (35,7% of the cases) (85). Rarely, blood can leak through the retroperitoneal space, causing swelling and a bluish discoloration of the scrotum, mimicking acute scrotal disease (80).

Since AH is usually unilateral, AI is infrequent. In a large series of 74 cases, AI was present in only 1 patient (1.3%) (81). AI usually develops during the first week of life, but delayed presentation may be due to gradual fibrosis of the adrenal gland or complications of PA, such as sepsis, clotting problems, and intraventricular hemorrhage.

The symptoms of AI are highly non-specific, so clinicians should keep a low threshold of suspicion for AI in patients with bilateral AH, hemodynamic instability, hypotension, lethargy, hypovolemic shock, hyponatremia, hyperkalemia, hypoglycemia, acidosis, or cholestasis (86). The development of AI is more frequent in preterm than in full-term babies (84). The adrenal gland has good regenerative capacity, so that complete regression of hemorrhagic lesions and even AI can be achieved within up to 30 months (45, 85, 87).

Laboratory work-up may variably show anemia, indirect hyperbilirubinemia, coagulation abnormalities, as well as hormonal hallmarks of primary AI (46).

US is the technique of choice for the screening and follow-up of AH in neonates and provides important clues for the differential diagnosis (**Table 2**) (45, 46), especially by documenting the typical changes in lesion appearance. Indeed, AH initially appears as a solid, echogenic lesion, but within 2 weeks it takes on a cystic appearance with mixed echogenicity and progressively resolves with possible residual calcifications (45). Contrast-enhanced US is more accurate than Doppler in differentiating non-vascularized AH lesions in equivocal cases (i.e. lesions with a constant or slow-progressing appearance over time) (87).

**Table 2:** Differential diagnosis of neonatal adrenal hemorrhage.

Adrenal gland
• Cyst • Abscess • Pulmonary sequestration • Neuroblastoma • Congenital adrenal hyperplasia • Lymphangioma • Myelolipoma • Metastases
Upper pole of the kidney
• Hydronephrosis • Multicystic dysplastic kidney • Double renal collecting system • Wilms’ tumor • Cystic nephroma

Some authors recommend abdominal US screening in all neonates with PA, especially in the presence of traumatic delivery, anemia, and prolonged jaundice (81). In patients receiving HT, routine abdominal US should be undertaken at the start of treatment and after the warm-up phase. When AH is identified, the kidneys must be scanned, to rule out concurrent renal vein thrombosis (88). Follow-up US is also required to evaluate lesion resolution. Although the timing is not well defined, a sensible approach might be to repeat the US every few days in the first 2 weeks, and then monthly until resolution.

Most cases of AH require only careful monitoring of vital signs, hydration, electrolytes and blood glucose, to allow early detection of AI (46). ACTH and Cortisol levels should be checked in all patients with bilateral AH at diagnosis and repeated regularly during the first month of life. AI is confirmed when baseline cortisol is <5 µg/dl (140 nmol/l), associated with ACTH concentrations more than two-fold the upper limit of the reference range, decreased aldosterone, and increased renin concentrations (47). If baseline values are borderline, a corticotropin test may be required, showing peak cortisol <18 µg/dl (500 nmol/l) 30 or 60 minutes after administration of ACTH (250 µg/m2 or 15 µg/kg). Basal cortisol concentrations >20 μg/dL exclude AI (46).

Treatment of acute AI is based on fluid, electrolyte, and hydrocortisone replacement (**Table 1**). Treatment cannot be delayed until confirmatory results are received and must be started immediately after a diagnostic sample is collected (46). Hydrocortisone is given as an initial iv bolus of 50-100 mg/m2, followed by 50-100 mg/m2/day in four divided doses. When the patient is clinically stable and able to take oral medications, hydrocortisone can be switched to an oral dose of 10-12 mg/m2/day in three divided doses and fludrocortisone replacement can be added. An attempt to gradually reduce hydrocortisone should be made when the patient is clinically stable, adrenal lesions are resolved or stable, and the hormone profile is repeatedly normal. Given the possible delayed resolution of AH, more than one attempt may be required (89).

#### Relative adrenal insufficiency

The hypothalamic-pituitary-adrenal axis acts as a key homeostatic regulator during PA, through a different pattern of release of cortisol, based on fetal characteristics and the severity and duration of hypoxic insult (90). In response to acute asphyxia and HT ovine fetuses exhibit a transient rise in cortisol, which is comparable in magnitude between preterm and full-term fetuses in case of severe injury (90). Moreover, study in humans demonstrated a more gradual decrease in cortisol concentration in neonates undergoing HT, compared to normothermic neonates, with cortisol values related to the anti-inflammatory cytokine Interleukin-10 (91). Despite such adaptations, some neonates with PA may experience hemodynamic instability and refractory hypotension (defined as a mean blood pressure persistently below the 10% percentile for age despite adequate inotropes and crystalloids administration), associated with relative adrenal insufficiency (48). This condition, also called critical-illness-related corticosteroid insufficiency (CIRCI), consists of cortisol secretion or activity that is inappropriately low for the extent of stress or severity of illness present (92). Thus, adrenal function must be evaluated in all neonates with symptoms of AI, regardless of the presence of AH. However, there are still very few studies conducted in neonates.

The mechanisms leading to CIRCI likely include impaired cortisol production (possibly related to reduced adrenal perfusion or impaired binding of ACTH to its adrenal receptor), as well as tissue resistance to glucocorticoids related to dysfunction of their receptors (84), with an impaired translocation of glucocorticoid receptors inside the cell nucleus in response to stress (93). Kashana et al. (94) found that half of the patients with HIE had circulatory collapse which improved with glucocorticoids administration, despite cortisol concentrations comparable to other asphyxiated neonates. Moreover, hypotensive babies had a marked increase in dehydroepiandrosterone suggesting selective impairment of the 3-beta-hydroxysteroid dehydrogenase enzyme activity during PA (94). In another study, asphyxiated neonates undergoing HT showed a CIRCI-compatible cortisol concentration at 24 hours of life, at least partially responsible for hypotension and multi-organ failure (91). Although neonates with refractory hypotension have an impaired response to ACTH stimulation and an inappropriately low cortisol level for the degree of stress, the diagnosis of CIRCI in this age group remains unclear, due to the lack of a specific cut-off value (49).

Administration of hydrocortisone or dexamethasone to neonates with refractory hypotension has been shown to improve blood pressure within 2 hours, mainly by acting on vascular tone (**Table 1**). Furthermore, it is worth mentioning that high doses of hydrocortisone in the murine model of HIE have shown some neuroprotective effects, especially in the presence of concomitant sepsis (95).

### Thyroid function

Thyroid hormones are key regulators of thermogenesis, water and electrolyte balance, and growth and development of the brain (96). Moreover, during asphyxia, they play important actions in regulating the cardiac contractile function (97, 98). Since the heart turns to anaerobic glycolysis for energy production during hypoxia and ischemia, triiodothyronin (T3) is important for the regulation of cellular heart metabolism. PA may be associated with a reduction of T4 and T3 levels and an increase in rT3, not caused by an intrinsic abnormality in thyroid function, especially over the first hours/days of life. This condition is known as euthyroid sick syndrome or, alternatively as non-thyroidal illness syndrome (NTIS) (99, 100). NTIS is more frequent in premature than in full-term newborns and may also be associated with severe diseases complicating PA, like respiratory distress, sepsis, cranial hemorrhage, persistent ductus arteriosus and necrotizing enterocolitis (99). Different pathogenic mechanisms have been hypothesized, such as abnormal setting of the hypothalamus and pituitary thyroid hormone receptor, asphyxia-induced changes in iodothyronine deiodinases expression and variations of intracellular thyroid hormone uptake (100, 101). The typical pattern of NTIS includes reduced T3 and increased rT3 concentrations, in the presence of low-normal TSH and suppressed response of TSH to thyrotropin-releasing hormone (TRH), while decreased T4 and FT4 concentrations are seen when the disease becomes more severe, correlating with poor prognosis (96–98). Tahirovic (96) and Sak (102) demonstrated lower cord blood FT4 and T4 concentrations in full-term babies with a low Apgar score compared to matched controls. Low serum concentrations of T4, FT4, T3 and FT3 have also been reported at 18 and 24 hours of life in asphyxiated newborns (103). Contrasting results for cord blood TSH concentrations in asphyxiated newborns have been reported. Sak (102) and Gemer (104) found higher values compared to the control group, possibly due to the catecholamine increase and/or redistribution of fetal blood flow to the brain. Conversely, Tahivoric (96) and Pereira (103) found no difference in cord blood TSH concentrations, thus leading to the inference of a low TRH secretion in response to hypoxia and/or stress. Alterations in thyroid function tests in asphyxiated newborns are likely to be influenced by TH, thus making the evaluation of thyroid function more complex. In adults, serum T3 and FT3 decrease when body temperature is low for a prolonged time (105). Studies evaluating thyroid hormones in infants who received HT have yielded contrasting results. In fact, while Yazici (97) found a higher capillary TSH in the first 4 days of life, Kobayashy et al. (64) reported TSH decrease to the lower limit of normal range at 24 hours, along with low serum FT3 and FT4 over the first 96 hours of life in asphyxiated newborns undergoing TH with abnormal MRI findings, compared with a group having normal imaging, suggesting central hypothyroidism associated with moderate/severe HIE.

Moreover, the use of certain drugs may contribute to altered thyroid function of asphyxiated neonates. In particular, the inotropic agent dopamine may inhibit TSH secretion by regulating gene expression and suppressing T4, acting directly on the thyroid gland (98, 106).

NTIS likely represents an adaptive response to stress in an attempt to reduce the metabolic rate and protect organs from illness-related hypercatabolism (106). In case of intact pituitary function, NTIS normalizes within 5 days from the hypoxic-ischemic event (107) or at least on discharge, following resolution of the acute disease (64).

Moreover, there is evidence that low serum thyroid hormone concentrations do not necessarily reflect tissue concentrations, which may depend upon the organ and type of insult (108). Therefore, NTIS does not usually require therapeutic intervention.

Although there is no convincing evidence regarding the usefulness of administering thyroid hormones to neonates with HIE, in case of moderate/severe HIE it is suggested to perform a thyroid function investigation at 72 hours or 96 hours of life, and before discharge, especially in hypoxic neonates who receive HT. Furthermore, according to current guidelines (109), screening of thyroid function must be repeated in acutely ill neonates at 2-4 weeks of life.

Substitutive L-thyroxine replacement treatment should be considered in cases of overt primitive congenital hypothyroidism or persistently low FT4 in the face of low-normal TSH concentrations, suggesting central hypothyroidism.

### Pineal function

The pineal gland, together with the suprachiasmatic nucleus, the hypothalamus, and reticulohypothalamic tract, play an important role in regulating the circadian rhythm and endocrine output, thus facilitating adaptation to environmental changes. However, its role in the context of HIE is still unclear. Studies on the murine model with HIE have shown dysregulated expression in the pineal gland of major clock genes, such as Clock and Bmal1 as yet as 48 hours after the hypoxic insult (110, 111), resulting from several mechanisms, including increased hypoxia-inducible factor (HIF)-1α and reactive oxygen species and/or activation of the hypothalamus-pituitary-adrenal axis (110). In neonates, disturbed expression of clock genes may mediate the decrease in brain metabolism during HIE, with reduced energy supply and neuronal death, and exert detrimental effects on cardiovascular function, coagulation, and immune system (111). Constant exposure of patients to artificial light in intensive care services may aggravate (112), while exogenous melatonin administration may ameliorate (113) such dysregulation.

Pineal cysts are frequent in MRI examinations (114). Laure-Kamionowska et al. (115) revealed hemorrhagic, necrotic, and cystic changes of the pineal glands in autopsied fetuses and newborns with other brain lesions, suggesting a high susceptibility to injury of the fetal and neonatal pineal gland parenchyma.

A key role of ischemic injury has been hypothesized in the pathogenesis of pineal cysts. Özment et al. (116) found that the prevalence of pineal cysts was higher than in healthy controls in term babies with periventricular leukomalacia likely due to hypoxic injury. Finally, Bregant et al. (117) found a prevalence of pineal cysts around 36% in adolescents born near-term who had HIE. Ischemic insult might lead to cavitation of the pineal gland due to damage from free radicals and toxins leading to necrotic degeneration of the intrapineal gliotic layer (115, 116).

Despite being mostly asymptomatic, pineal cysts have been associated with apoplexy, precocious puberty and headache (118). Furthermore, reduced production of melatonin decreases the infants’ resistance to various harmful environmental agents and could be related to psychomotor retardation (119). Indeed, melatonin receptors have been found in central and peripheral tissues from the early stages of intrauterine growth (120), when melatonin plays important roles in implementing the genetic program for the development of the brain and other organs (121). In addition, this hormone and its metabolites regulate biological rhythms and acts as a potent endogenous anti-inflammatory, anti-apoptotic and antioxidant agent directly or indirectly, by inducing the synthesis of antioxidant enzymes (122). Indeed, chronic hypoxia may induce adaptations in the fetal metabolism of tryptophan and serotonin (precursors of melatonin) involved in the regulation of synaptogenesis, to prevent inflammation and neuronal death (123, 124).

Melatonin is currently recommended only for regulating sleep-wake rhythm (125); preclinical studies have shown additive neuroprotective effects to hypothermia, allowing for reduction in infarct size and preservation of neurons (126, 127). A recent randomized placebo-controlled trial (128) confirmed the positive effects of intravenous melatonin to infants in the early phase of HIE on cognitive outcome at 18 months of life, with a good safety profile. Further studies are needed to clarify the benefits of melatonin in HIE and to assess the efficacy of enteral administration.

### Pituitary function

To our knowledge, there are no reports in the literature of anterior pituitary deficits developing during the early course of perinatal asphyxia or during HT. Indeed, in other periods of life pituitary ischemic necrosis mainly occurs when the gland is enlarged by a tumor or non-tumor process, which disrupts vascular microarchitecture and increases blood supply requirement (69). Nevertheless, pituitary defects potentially related to PA may occur beyond the immediate postnatal period, highlighting the need for long-term follow-up of asphyxiated infants, including regular evaluation of growth and pubertal development.

#### Growth hormone deficiency

Several but not all studies (129) have reported a higher prevalence of PA among children with isolated growth hormone deficiency (GHD) or multiple pituitary hormone deficiency (MPHD) than in the healthy population (130–133), even though prevalence data vary greatly between studies.

An Italian study reported that 19/48 (39.6%) children with GHD had a perinatal history of breech delivery and/or prolonged asphyxia (134). A similar prevalence of asphyxia (15/42, 36%) was also reported in Japanese patients with idiopathic GHD (135). Conversely, Dasai et al. reported PA in only 7/75 (9.3%) children with idiopathic GHD (136), while a Japanese survey conducted from 1986-1998 on 23,110 patients with idiopathic GHD found a prevalence of asphyxia at delivery of 12.3% (137). Interestingly, children with more severe growth hormone deficiency showed the highest prevalence of PA (up to 21.8%) (137). More recently, another large series of 19,717 Japanese children with GHD treated from 1996 to 2015 (138) reported a prevalence of asphyxia of 6.9%, with a gradual decline in prevalence over the study span. Taken together, these observations have questioned whether PA can be considered a cause of hypothalamic pituitary dysfunction and/or GHD later in life in some cases defined as idiopathic.

Birth asphyxia has also been associated with growth impairment in children with pre-dialysis chronic kidney disease, suggesting that a history of asphyxia could help clinicians in identifying those children who can most benefit from timely GH treatment (139).

The mechanisms underlying the association between PA and GHD are still poorly understood. Magnetic resonance imaging (MRI) studies (140, 141) have shown a higher prevalence of history of PA or breech delivery in GHD patients with ectopic posterior pituitary, compared with individuals with normal pituitary gland, reaching 100% in the case of severe isolated GHD (141) or MPHD (142). These findings have led to the hypothesis that pituitary abnormalities and dysfunction may arise from a traumatic-ischemic insult.

Finally, it is worth mentioning that a role for GH in mitigating neurodevelopmental sequelae of PA has also been postulated. Devesa et al. reported in a 10-year-old girl with PA without GHD that GH treatment associated with neurorehabilitation significantly increased cognitive abilities, memory, language competence index, and IQ score (143). This data, even if referred to a single case and associated with the rehabilitation treatment, is in line with the positive effects of GH on neurocognition observed in other conditions, such as Prader Willi syndrome (144).

#### Central precocious puberty

Central precocious puberty (CPP) may result from acquired brain abnormalities, including neonatal HIE and CP (145–148). Previous data documented that children with a neurodevelopmental disability are 20 times more at risk of premature pubertal changes than the general population (149). In a prospective study of 161 girls with HIE (150), early sexual maturation was documented in 4.3% of cases (almost 7 folds more than in the general population). Interestingly, about half of girls with early puberty had no physical disability (150). Although the exact mechanisms by which brain lesions not involving the hypothalamus may trigger CPP is not known, it has been hypothesized that severe brain damage and the use of antiepileptic medication may affect several neurotransmitters pathways involved in the control of gonadotropins by inducing an activation of the HPG-axis (151, 152). Worley et al. evaluated 207 children with CP and demonstrated that both girls and boys appeared to enter puberty earlier than the general population, although the formers tended to mature over a longer period of time while the latters followed more regular patterns (148). More recent data from a retrospective, case-control study comparing pubertal patterns of children with CPP and CP with two other groups with CP without CPP, and CPP without CP, confirmed that CPP in CP seems to progress rapidly, supporting the hypothesis of a more intense activations of the HPG-axis (146). Moreover, blunted growth can make the diagnosis of CPP more difficult in patients with CP (146).

### Adipokines

Data regarding the role of adipokines during PA are scanty. In a recent study, El Mazari et al. (28) reported lower concentrations of adiponectin, and higher concentrations of leptin, compared to healthy controls, which were not related to anthropometric parameters or insulin concentrations as normally observed. Such results may reflect hypoxia-related adipose tissue damage, peripheral tissue resistance or alteration of the endocrine, paracrine, and autocrine mechanisms that control adipokine release (28). These metabolic changes may represent a metabolic adaptation to hypoxia. Indeed, *in vitro* and *in vivo* studies have shown neuroprotective effects of leptin in ischemic brain injury, consisting in increased neuronal density and reduced apoptosis (153–155).

## Conclusions

The implications of PA and HT from the endocrine perspective are not yet well defined. The relationship between PA and the endocrine systems is multifaceted. Indeed, if on one side PA is normally accompanied by a marked endocrine and paracrine neuroendocrine response, on the other side hypoxic-ischemic injury, as well as the failure of the compensatory physiological mechanisms may lead to several endocrine complications. HT exerts only partial neuroprotective effects and may in turn cause endocrine derangement. Therefore, it is necessary to think about alternative or additive strategies improving neuroprotection are desirable.

Noteworthy, some manifestations (ie hypercalcemia, GHD and CPP) may occur beyond the immediate postnatal period, highlighting the need for long-term follow-up of asphyxiated infants. Given the delicate balance between the various medical conditions possibly occurring in the context of PA and HT, a multidisciplinary approach is desirable to identify the best case-by-case management. Adequate monitoring of several endocrine functions and prevention of secondary injury by ensuring optimal glucose and electrolytes homeostasis are essential to improve outcome and prevent life-threatening events.

## Author contributions

Conceptualization: NI, DC, and MS. Original draft preparation: NI, LS, MC, LL, GTu, LS, DT, JM, LP, FT, MP, FV, TA, GG, AP, MV, and GTr. Review and editing: MS, LS, DC, and TA. Visualization: NI, MS, DC, and TA. All authors contributed to the article and approved the submitted version.
